# Antimicrobial resistance awareness and food safety concerns among rural households in Zimbabwe

**DOI:** 10.1016/j.onehlt.2025.101208

**Published:** 2025-09-15

**Authors:** George Kembo, Lesley Macheka, Mavis P. Dembedza, Desmond T. Mugadza, Faith A. Manditsera

**Affiliations:** a Food and Nutrition Council of Zimbabwe, 1574 Alpes Road, Hatcliffe, Harare, Zimbabwe; b Directorare for Innovation, Industrialisation and Enterprise Development, University of Agricultural Sciences and Technology, P. O. Box 35, Marondera, Zimbabwe; c Research and Innovation Directorate, Great Zimbabwe University, P. O. Box 1235, Masvingo, Zimbabwe; d Department of Food Processing Technology, Harare Institute of Technology, Ganges Road, BE 277, Belvedere, Harare, Zimbabwe.

**Keywords:** Antimicrobial resistance, Food safety, Behaviour, One health, Rural households Zimbabwe

## Abstract

Antimicrobial resistance (AMR) and unsafe food management are important yet overlooked problems in rural sub-Saharan Africa. This study was aimed at assessing the level of awareness and knowledge regarding antibiotic use and withdrawal periods and understanding the factors that influence AMR awareness in rural Zimbabwe. Data was gathered from 17,895 households in all 60 rural districts in Zimbabwe through a household survey. Descriptive statistics and adjusted Ordinary Least Squares (OLS) regression models were used to identify predictors of AMR awareness and safe food handling behaviours. Findings revealed limited (18.3 %) awareness of AMR, with notable disparities across education levels and religious affiliations. Higher educational attainment and wealth status were positively associated with both AMR awareness and food safety knowledge. On the other hand, households affiliated with conservative religious groups or those with larger family sizes demonstrated significantly lower adherence to recommended practices. The study underscores the intersection of behavioural, social, and structural determinants in shaping food safety and AMR-related behaviours. The findings provide vital empirical evidence to inform national strategies under the One Health framework and support efforts to reduce foodborne illness and antimicrobial misuse in low-income rural settings.

## Highlights

• Low AMR awareness (18.3 %) among rural households in Zimbabwe.
• Poor adherence to antibiotic withdrawal periods in livestock products.
• Education and wealth strongly predict AMR awareness and safe practices.
• Religious affiliation influences AMR knowledge and compliance behaviours.
• One Health approach critical for integrated AMR and food safety strategies.

## 1 Introduction

Antimicrobial resistance (AMR) has emerged as one of the most urgent global public health threats of the 21st century, reversing decades of progress in the treatment of infectious diseases in human and animal health. Overuse and misuse of antibiotics in agriculture, livestock, and human medicine have contributed significantly to the increased emergence of resistant microorganisms, with diminished treatment options and high risk for food safety [1,20]. In 2019, direct deaths attributed to AMR were estimated at more than 1.27 million people worldwide, with the highest burden being in sub-Saharan Africa [2,27]. Low-income and middle-income countries (LMICs) are most affected due to deficient surveillance systems, poor regulation, and poor access to health and veterinary services [12,38].

In Zimbabwe, as in many other LMICs, rural livestock plays a significant role in livelihoods, in terms of nutrition, income, and social status [24,37]. Unfortunately, weaknesses in terms of control over the use of antibiotics, which is mostly through self-medication of drugs mainly bought from informal markets, is associated with a high possibility of food contamination by antimicrobial residues [2]. The use and misuse of antimicrobial in livestock production is one of the major cause leading to increasing antimicrobial resistance [19]. Most rural households are usually poorly educated about the responsible use of antimicrobials [17,29], and knowledge on the importance of withdrawal times after treatment is limited [35], increasing the risk of unsafe animal product consumption. Further complicating this situation is the lack of trained personnel, contributing to dependence on traditional or non-expert advice, and promoting misuse of antibiotics in animal husbandry [7].

Food safety concerns arising from AMR are related to the consumption of meat, milk, or eggs, which retain antimicrobial residues. These residues may lead to allergic reactions, disrupt gut microbiota, and contribute to the development of resistant infections in humans [34]. Although the Codex Alimentarius contains global guidelines for monitoring antimicrobial residues, most LMICs, Zimbabwe included, do not have vigorous surveillance systems that ensure compliance [21,26], especially in informal markets that dominate food distribution in rural and peri-urban areas [11]. The lack of visible residues and lack of uniform food safety control measures for consumers reinforce requirements for awareness programmes and point-of-sale education. One Health has been recognised as a key cross-cutting approach addressing AMR by acknowledging the human-animal-environment interface [9]. It encourages a multisectoral approach to surveillance, governance, and behavioural interventions. The One Health approach incorporated in the Zimbabwe National Action Plan on AMR (2017–2021) was aligned with the global and regional strategies, which include the WHO Global Action Plan, FAO's Action Plan on AMR, and the World Organization for Animal Health (WOAH) strategy [22,39,42]. However, its effectiveness has been constrained due to insufficient funding, lack of coordination, and failure to decentralise services to access rural households [42].

Multiple global initiatives have been implemented in response to AMR through research support, technical capacity building, and stakeholder mobilisation [3,41,44]. Despite these efforts, major gaps persist in understanding AMR-related behaviours at household level. For example, rural livestock-keeping households are often excluded from mainstream AMR awareness campaigns. More so, cultural, religious, economic, and demographic factors influence antimicrobial use and food safety practices, yet these dimensions are rarely integrated into AMR strategies [5,31]. This study therefore aimed to assess the level of AMR awareness among rural households in Zimbabwe and identify factors understanding the factors that influence AMR awareness, contributing to the limited empirical evidence on AMR-food safety nexus. Findings from this study will inform policies and programmes that promote responsible antimicrobial use contributing to improved food safety, and strengthen the One Health agenda for AMR containment in Zimbabwe and comparable LMIC settings

## 2 Methodology

### 2.1 Sampling

Data was collected from all 60 rural districts in Zimbabwe from 21 May 2025 to 4 June 2025. The sampling strategy was based on the Zimbabwe National Statistics Agency (ZimSTAT)’s master sampling frame. A multi-stage cluster sampling technique was adopted to select a representative sample of households in all rural districts. This approach allowed for probability-based selection at multiple levels, district, ward, enumeration area (EA), and household, thereby minimising selection bias and ensuring that all households had a known, non-zero chance of selection. Within each district, wards were selected using Probability Proportional to Size (PPS), meaning wards with larger populations had a higher chance of being selected. Within each selected ward, enumeration areas (EAs) were also selected using PPS. A minimum sample size of 300 households was calculated using the Cochran formula [8] with 95 % confidence level and 5 % margin of error, resulting in a total of 17,895 households included in the study. After excluding incomplete responses, 17,785 households were included in the AMR knowledge model A sub-sample of 3940 livestock-owning households was analysed for practices related to antibiotic use and withdrawal periods.

### 2.2 Data collection protocols

A set of standardised protocols guided the administration of data collection tools to ensure consistency and data reliability. A structured questionnaire, pre-tested with 10 households for validity and reliability, and was used to collect data on demographic variables, AMR awareness, and food safety practices through face-to-face interviews. Knowledge of AMR was assessed using the question: ‘Have you ever heard of antimicrobial resistance (sometimes called antibiotic resistance)?’ Respondents who answered ‘yes’ and could describe it further were coded as ‘aware’; all others were coded as ‘not aware’. More so, for the purposes of this study, the withdrawal period was defined as the recommended interval between the last administration of an antimicrobial and the safe consumption of animal products. In Zimbabwe, this typically ranges from 3 to 7 days for milk and eggs, and 7–28 days for meat, depending on the drug used. These intervals were explained to respondents during interviews.

A total of 120 enumerators were deployed in 30 teams, each supervised by a field supervisor. Teams completed an average of 10–12 households per day, enabling coverage of all 60 districts within the two-week period. Data were collected using GPS-enabled tablets that allowed real-time monitoring. Spot checks and 10 % back-checks were conducted to ensure data quality and adherence to protocol. The study was conducted ethically following the World Medical Association Declaration of Helsinki [46] and informed consent was obtained from all participants prior to data collection In addition, the study received ethical approval from Marondera University of Agricultural Sciences and Technology (Ref: MUAST/07/2025).

### 2.3 Data analysis

#### 2.3.1 Analytical model used in the study

This study utilised STATA 15 as the statistical software. We employed an Ordinary Least Squares (OLS) regression model [45] to evaluate how background characteristics affect use of antibiotics, AMR awareness. To ensure the best estimates from OLS, several assumptions must be satisfied, including the one of constant error variance (i.e., homoskedasticity) [4]. The OLS regression model use is specified as:

(1) $$Y_{i}=\beta_{0}+\beta_{1}X_{1}+\beta_{2}X_{2}+\ldots +\beta_{i}X_{i}+e_{i}$$

where *Y*_*i*_ is the dependent variable, *X*_*i*_ is a vector of independent variables; *β*_0_ and *β*_*i*_ are the vectors of parameters to be estimated, and *e*_1_ is the error term.

All analyses incorporated survey weights, primary sampling units, and strata based on the ZIMSTAT sampling frame. This approach ensures that results are nationally representative and accounts for design effects inherent in the PPS cluster sampling

### 2.4 Study limitations and strengths

The cross-sectional nature of the study restricts the ability to make causal inferences concerning characteristics observed here and food safety outcomes. Additionally, the use of self-reported measures to assess the dependent and predictor variables creates a possibility for bias, as respondents might have exaggerated their positive attitudes or disclosed less undesirable ones. Notwithstanding these limitations, our study has some significant strengths. The study is one of the most extensive ones that is being conducted, a nationally representative survey of food safety and food safety practices in rural Zimbabwe, including 17,895 households in each of the 60 rural districts. Reliance on rigorous statistical techniques and a multi-stage probability sampling design further increases the credibility and generalisability of the findings. Furthermore, using sociodemographic, cultural, and health variables, the study provides nuanced perspectives essential for guiding initiatives and policies for tailored interventions and policies toward food safety and SDGs.

## 3 Results

### 3.1 Background characteristics

The analysis included data from a national sample of 17,895 households in Zimbabwe. Before controlling for unconfounding factors, the results in Table 1 show that the mean age of the household head was 48.88 years, with a standard deviation of 17.28, indicating a predominantly middle-aged sample population. Most of the households were male-headed (64.4 %), with 35.6 % being female-headed households. In terms of educational attainment, most household heads had either completed O′ Level (37.0 %) or attained only a primary-level education (34 %). Only 2 % had attained advanced to A' Level or higher education, and only about 0.6 % had obtained post-secondary qualifications. Regarding marital status, 60.2 % of household heads were married and living together, while 10.1 % were married but living apart. Widowed household heads constituted 19.7 %, and 7.4 % were divorced or separated. Religious affiliation was heavily skewed toward Apostolic Sect members, who comprised 37.3 % of respondents. This was followed by Pentecostal (11.5 %), Protestant (7.6 %), and Zionist (10.8 %) affiliations. At least 15.4 % of household heads reported having no religious affiliation. Furthermore, the results indicate that mean household size was 3.89 individuals, and 6.8 % of households had a member who is disabled. Chronic illness was reported in 15.2 % of the sample, and the average asset index was 6.36, indicating moderate socio-economic variation across households. The relatively high standard deviations observed in several variables reflect the heterogeneity of rural households across provinces and socio-economic groups, which is expected in a nationally representative survey of 17,895 households.

**Table 1:** Descriptive statistics for background characteristics.

Variable	Mean (SD)
Age (years)	48.88 (17.28)
Male-headed households	0.64 (0.48)
Female-headed households	0.36 (0.48)
**Education level**	
None	0.08 (0.28)
Primary	0.34 (0.47)
ZJC	0.17 (0.38)
O′ Level	0.37 (0.48)
A' Level	0.01 (0.12)
Diploma/certificate after primary	0.01 (0.08)
Diploma/certificate after secondary	0.01 (0.10)
Graduate/postgraduate	0.01 (0.08)
**Marital status**	
Married, living together	0.60 (0.49)
Married, living apart	0.10 (0.30)
Divorced/separated	0.07 (0.26)
Widowed	0.20 (0.40)
Cohabiting	0.00 (0.06)
Never married	0.02 (0.15)
**Religion**	
Roman Catholic	0.07 (0.26)
Protestant	0.08 (0.27)
Pentecostal	0.12 (0.32)
Apostolic Sect	0.37 (0.48)
Zion	0.11 (0.31)
Other Christian	0.06 (0.24)
Islam	0.00 (0.06)
Traditional	0.02 (0.15)
Other religion	0.01 (0.12)
No religion	0.15 (0.36)
**Household characteristics**	
Household size	3.89 (1.71)
Household head disability	0.07 (0.25)
Chronic illness in household	0.15 (0.36)
Asset index	6.36 (3.39)
**Province**	
Manicaland	0.12 (0.32)
Mashonaland Central	0.13 (0.34)
Mashonaland East	0.15 (0.36)
Mashonaland West	0.12 (0.32)
Matabeleland North	0.12 (0.32)
Matabeleland South	0.12 (0.32)
Midlands	0.13 (0.34)
Masvingo	0.12 (0.32)

### 3.2 Descriptive statistics

#### 3.2.1 Knowledge of AMR and associated unsafe meat consumption practices

Findings presented in Table 2 indicate that national awareness of antimicrobial resistance was relatively low, with only 18.3 % of households reporting any knowledge of the concept. The highest awareness was observed in Mashonaland Central (25.3 %), Matabeleland North (23.2 %), and Masvingo (22.3 %), while the lowest was reported in Mashonaland West (8.2 %). These findings indicate substantial provincial disparities and underscore the need for targeted awareness campaigns. Furthermore, Table 2 reveals that 5.7 % of households had consumed meat from animals that were known to be on antibiotic treatment and had not yet completed the prescribed withdrawal period. The provinces of Masvingo (8.2 %) and Midlands (7.7 %) reported the highest levels of such behaviour, which poses a significant risk of antimicrobial residues entering the human food chain, compromising food safety.

**Table 2:** Knowledge of Antimicrobial Resistance and adherence to the withdrawal periods to pesticides and antimicrobial resistance.

Province	Households aware of AMR (%)	Households aware of AMR (%)
Manicaland	20.0	4.5
Mashonaland Central	25.3	4.8
Mashonaland East	16.5	5.4
Mashonaland West	8.2	5.0
Matabeleland North	23.2	7.0
Matabeleland South	15.0	2.5
Midlands	15.5	7.7
Masvingo	22.3	8.2
**National**	**18.3**	**5.7**

#### 3.2.2 Use of antibiotics and practices

The results presented in Table 3 show the use of antibiotics and associated practices among surveyed households in relation to livestock treatment. The findings indicate significant trends in the levels of awareness and compliance with best practices, especially concerning the use of antibiotics, adherence to withdrawal periods, and duration of treatment. Overall, the data shows a consistent pattern of limited routine or proper use of antimicrobials and poor adherence to best practices, suggesting potential public health and food safety risks.

**Table 3:** Use of antimicrobials and practices among livestock-owning households (%) (*n* = 3940).

***Variable***	**Responses (%)**				
	Never	Rarely	Sometimes	Often	Always
1. *Households use antibiotics to treat their livestock*	93.1	3.2	2.3	0.6	0.9
2. Households read the instructions regarding withdrawal periods when using medicine to treat their livestock	90.9	3.0	2.6	1.0	2.5
3. *Households continue giving the livestock medication for the full prescribed duration, even when they no longer appear sick*	92.5	2.8	1.9	0.9	1.9
4. Households observe the prescribed withdrawal periods before selling the livestock treated with antibiotics	91.6	2.3	1.7	1.3	3.1
5. Households follow the prescribed withdrawal period before consuming meat, milk and eggs from treated livestock	89.9	2.6	2.1	1.6	3.8

#### 3.2.3 Use of antibiotics to treat livestock

Most (93.1 %) of the households reported that they *never* use antibiotics to treat their livestock, while 0.9 % reported *always* using antibiotics, and only 0.6 % do so *often*. A small proportion reported occasional use, with 3.2 % using them *rarely* and 2.3 % *sometimes*. These results could indicate limited access to veterinary pharmaceuticals or low awareness of their role in animal health. However, the infrequent use may also reflect informal practices, under-reporting, or reliance on traditional or non-pharmaceutical treatments, raising concerns about untreated or improperly managed livestock diseases.

#### 3.2.4 Reading instructions on withdrawal periods

When asked whether the households read instructions related to withdrawal periods (the time required after drug administration before animal products can be safely consumed or sold), 90.9 % of households reported they *never* read such instructions. Only 2.5 % *always* read them, while 1.0 % reported *often* doing so. This low level of compliance suggests a serious knowledge gap regarding drug residue risks in animal-source foods. The small percentage of households who *rarely* (3.0 %) or *sometimes* (2.6 %) read instructions underscores the need for targeted education and clearer communication of withdrawal guidelines at the point of sale or treatment.

#### 3.2.5 Completing the full prescribed medication duration

A high proportion of households (92.5 %) indicated that they *never* continue giving livestock antibiotics for the full prescribed course once the animals appear to have recovered. Only 1.9 % adhere *always*, and another 0.9 % do so *often*. The rest either *rarely* (2.8 %) or *sometimes* (1.9 %) complete the treatment. This finding is particularly concerning as it reflects poor compliance with treatment regimens, which can contribute to the development of antimicrobial resistance, a major global health threat. This result also suggests a lack of veterinary oversight and inadequate understanding of why full treatment courses are critical.

#### 3.2.6 Observing withdrawal periods before selling treated livestock

Regarding observing withdrawal periods before selling livestock treated with antibiotics, 91.6 % of households reported they *never* do so. A very small percentage *always* observe the withdrawal period (3.1 %), while 1.3 % report doing so *often*. This indicates that a significant portion of livestock being sold in the market may contain antibiotic residues, posing risks to consumer health and contributing to antimicrobial resistance. The lack of compliance can be attributed to poor regulation enforcement, market pressures for income, or lack of knowledge on the implications of not observing the withdrawal period.

#### 3.2.7 Observing withdrawal periods before consumption of animal products

Most of the households (89.9 %) indicated they *never* observe the withdrawal period before consuming meat, milk, or eggs from treated animals. Only 3.8 % *always* observe the waiting period, while others report *often* (1.6 %), *sometimes* (2.1 %), or *rarely* (2.6 %). This is a critical finding as it reveals that a large segment of rural households may be exposing themselves to veterinary drug residues through self-consumed livestock products. This practice can compromise food safety, impact health outcomes, and contribute to long-term antimicrobial resistance risks in the population. Overall, the results in Table 3 reveal a significant deficiency in knowledge and practices related to antimicrobial use and withdrawal period observance among livestock-owning households. While antibiotic use itself appears low, which might on the surface seem positive from an AMR standpoint, the associated practices around reading instructions, completing treatment, and adhering to withdrawal periods are notably inadequate.

### 3.3 Regression analysis

#### 3.3.1 Predictors of knowledge on antimicrobial resistance

Table 4 presents the regression model estimating the likelihood of having knowledge about antimicrobial resistance. After controlling for confounding factors, educational attainment was a strong predictor as households whose heads had completed primary (coef = 0.035, *p* < 0.01), ZJC (coef = 0.029, *p* < 0.05), O′ Level (coef = 0.034, *p* < 0.01), A' Level (coef = 0.097, p < 0.01), or a diploma after secondary school (coef = 0.083, p < 0.05) had significantly higher likelihood of knowing about AMR, compared to those with no formal education. Religion was also an important factor. Protestant (coef = 0.044, *p* < 0.01) and Pentecostal (coef = 0.026, *p* < 0.1) households were more likely to be knowledgeable about AMR. Furthermore, households with a member living with disability were more likely to be knowledgeable about AMR (coef = 0.052, *p* < 0.01), while those with chronic conditions were less likely to report such knowledge (coef = −0.029, p < 0.01). Wealth, as measured by the asset index, was positively associated with AMR knowledge (coef = 0.015, p < 0.01), confirming that socio-economic status influences access to information. Segregating the findings by province, households located in Mashonaland Central (coef = 0.056, p < 0.01) and Matabeleland North (coef = 0.024, *p* < 0.1) provinces had significantly higher AMR awareness, whereas those in Mashonaland West, Midlands, and Matabeleland South provinces had significantly lower awareness.

**Table 4:** Predictors of household knowledge about antimicrobial resistance (*n* = 17,785).

**Variable**	**Coef.**	**SE**	***p*-value**
Household head age	0.000	0.000	0.42
Female-headed household	−0.011	0.009	0.22
**Education (ref: none)**			
Primary	0.035	0.010	0.001
ZJC	0.029	0.012	0.019
O′ Level	0.034	0.011	0.002
A' Level	0.097	0.030	0.001
Diploma after secondary	0.083	0.034	0.014
Graduate/postgraduate	0.050	0.043	0.25
**Religion (ref: Roman Catholic)**			
Protestant	0.044	0.016	0.006
Pentecostal	0.026	0.014	0.072
Apostolic Sect	0.009	0.012	0.46
Zion	0.011	0.014	0.43
Other Christian	−0.026	0.015	0.08
Islam	−0.077	0.038	0.04
Other religion	0.059	0.029	0.04
**Household characteristics**			
Household size	−0.003	0.002	0.09
Disability	0.052	0.012	0.001
Chronic illness	−0.029	0.008	0.001
Asset index	0.015	0.001	0.001
**Province (ref: Manicaland)**			
Mashonaland Central	0.056	0.013	0.001
Mashonaland East	−0.034	0.011	0.002
Mashonaland West	−0.109	0.011	0.001
Matabeleland North	0.024	0.013	0.07
Matabeleland South	−0.048	0.013	0.001
Midlands	−0.036	0.012	0.003
Masvingo	0.015	0.013	0.25

Survey-adjusted SEs in parentheses. *** p < 0.01, ** p < 0.05, * *p* < 0.1.

#### 3.3.2 Predictors of antibiotic use and compliance in livestock management

Table 5 shows the results on predictors of antibiotic use in livestock and compliance with AMR-related practices by rural households in Zimbabwe. The results reveal that older household heads were more likely to use antibiotics (coef = 0.002, *p* < 0.05) in treating their livestock and observe withdrawal periods before selling treated animals (coef = 0.002, p < 0.05). However, higher levels of education showed mixed results. Graduate and postgraduate-level education was negatively associated with antibiotic use (coef = −0.129, *p* < 0.01) and continuing medication when symptoms disappeared (coef = −0.120, p < 0.05), potentially indicating more selective or informed antibiotic use. Religious affiliation had a significant impact, households affiliated with the Zion Church or classified under “Other Christian” had higher compliance with continuing prescribed medication and observing withdrawal periods before consumption and sale. Cohabiting households consistently showed lower compliance across all models, suggesting that household structure may influence food safety behaviour. More so, geographical variation was evident as rural households in Matabeleland North had significantly higher adherence across multiple indicators, while Mashonaland West and Matabeleland South were consistently associated with poor compliance. Higher household wealth was a positive predictor of compliance with withdrawal instructions and antibiotic use.

**Table 5:** Regression analysis for livestock practices/consumption with background characteristics.

	Use antibiotics to treat your livestock		Read the instructions regarding withdrawal periods when you use medicine to treat		Continue giving the livestock medication for the full prescribed duration even		Observe the prescribed withdrawal periods before selling the livestock treated w		Follow the prescribed withdrawal period before consuming meat	
VARIABLES	coef	Se	coef	se	coef	se	coef	se		se
Household head age	0.002	(0.001)**	0.001	(0.001)	0.002	(0.001)*	0.002	(0.001)**	0.001	(0.001)
Female-headed household	−0.028	(0.031)	−0.024	(0.040)	−0.021	(0.037)	−0.020	(0.044)	0.019	(0.047)
Primary level	0.011	(0.037)	0.011	(0.051)	0.025	(0.045)	0.008	(0.058)	−0.002	(0.062)
ZJC level	0.027	(0.041)	0.006	(0.054)	0.025	(0.048)	−0.000	(0.061)	−0.041	(0.065)
O′ level	0.019	(0.039)	−0.015	(0.052)	0.022	(0.048)	−0.034	(0.060)	−0.064	(0.064)
A' level	0.111	(0.087)	0.179	(0.129)	0.164	(0.119)	0.098	(0.129)	0.042	(0.135)
Diploma/Certificate after primary	−0.143	(0.049)***	0.156	(0.225)	0.033	(0.124)	−0.020	(0.182)	0.028	(0.203)
Diploma/Certificate after secondary	−0.075	(0.051)	0.044	(0.166)	0.086	(0.146)	−0.108	(0.133)	−0.183	(0.136)
Graduate/postgraduate	−0.129	(0.047)***	−0.060	(0.137)	−0.120	(0.054)**	−0.127	(0.118)	−0.013	(0.198)
Married living apart	0.013	(0.035)	−0.013	(0.046)	−0.048	(0.039)	−0.019	(0.050)	−0.070	(0.055)
Divorced/separated	0.034	(0.040)	0.008	(0.050)	−0.031	(0.045)	−0.013	(0.054)	−0.059	(0.059)
Widow/widower	−0.005	(0.042)	−0.007	(0.057)	−0.018	(0.052)	−0.029	(0.062)	−0.074	(0.067)
Cohabiting	−0.102	(0.028)***	−0.167	(0.037)***	−0.174	(0.035)***	−0.187	(0.039)***	−0.247	(0.041)***
Never married	0.096	(0.063)	0.145	(0.083)*	0.154	(0.080)*	0.135	(0.084)	0.132	(0.090)
Protestant	0.082	(0.064)	0.205	(0.087)**	0.216	(0.076)***	0.138	(0.090)	0.175	(0.100)*
Pentecostal	−0.065	(0.036)*	−0.030	(0.050)	0.029	(0.038)	−0.034	(0.060)	−0.015	(0.066)
Apostolic Sect	0.005	(0.036)	0.053	(0.046)	0.088	(0.033)***	0.028	(0.057)	0.031	(0.061)
Zion	0.063	(0.050)	0.162	(0.066)**	0.143	(0.051)***	0.134	(0.077)*	0.188	(0.085)**
Other Christian	0.071	(0.053)	0.079	(0.063)	0.136	(0.053)***	0.079	(0.073)	0.082	(0.079)
Islam	0.003	(0.101)	−0.071	(0.097)	0.088	(0.174)	−0.031	(0.180)	−0.067	(0.183)
Traditional	0.053	(0.061)	0.187	(0.108)*	0.240	(0.105)**	0.142	(0.119)	0.162	(0.123)
Other religion	0.187	(0.148)	0.122	(0.153)	0.213	(0.147)	0.096	(0.155)	0.065	(0.156)
No religion	−0.009	(0.039)	−0.002	(0.049)	0.031	(0.035)	−0.036	(0.059)	−0.032	(0.064)
Household size	−0.006	(0.007)	0.007	(0.009)	−0.003	(0.008)	0.004	(0.010)	0.001	(0.011)
Household head disability	0.061	(0.055)	0.098	(0.072)	0.098	(0.067)	0.096	(0.079)	0.069	(0.084)
Household head chronic condition	0.020	(0.031)	−0.012	(0.038)	0.017	(0.035)	0.020	(0.043)	−0.002	(0.046)
Asset index	0.010	(0.004)***	0.011	(0.005)**	0.006	(0.004)	0.012	(0.006)**	0.010	(0.006)
Mashonaland Central	0.009	(0.032)	−0.064	(0.051)	−0.036	(0.048)	−0.075	(0.057)	−0.170	(0.063)***
Mashonaland East	0.021	(0.036)	−0.034	(0.054)	0.008	(0.053)	−0.027	(0.060)	−0.101	(0.067)
Mashonaland West	−0.037	(0.030)	−0.112	(0.052)**	−0.120	(0.047)**	−0.130	(0.056)**	−0.186	(0.065)***
Matabeleland North	0.120	(0.046)***	0.141	(0.071)**	0.057	(0.061)	0.126	(0.077)	0.008	(0.082)
Matabeleland South	−0.102	(0.033)***	−0.135	(0.057)**	−0.192	(0.049)***	−0.180	(0.060)***	−0.273	(0.069)***
Midlands	0.041	(0.037)	−0.079	(0.054)	−0.046	(0.050)	−0.081	(0.061)	−0.155	(0.070)**
Masvingo	0.069	(0.048)	0.006	(0.068)	−0.021	(0.059)	−0.051	(0.069)	−0.134	(0.077)*
Constant	0.996	(0.074)***	1.066	(0.100)***	1.026	(0.088)***	1.106	(0.118)***	1.295	(0.126)***
Observations	3940		3940		3940		3940		3940	
R-squared	0.026	0.025		0.026		0.022		0.020		

Robust standard errors in parentheses *** p < 0.01, ** p < 0.05, * p < 0.1.

## 4 Discussion

Despite the growing global concern on AMR, knowledge of AMR among Zimbabwean rural households remains low, with only 18.3 % reporting awareness. These findings are consistent with earlier research in sub-Saharan Africa, where awareness of AMR among the general population has been reported as low due to limited access to information, lack of health education, and poor integration of AMR messages into primary healthcare and agricultural extension [14,23,25]. In rural settings, particularly, AMR communication is often fragmented and not mainstreamed into routine livestock or human health outreach programmes, further exacerbating the challenge [10]. According to Kallu, S.A. [15] and Sadiq, M.B. [36], low awareness impairs informed decision-making around the use of antimicrobials, contributing to misuse and overuse, both of which are key drivers of resistance.

In addition, the study reveals low compliance with prescribed withdrawal periods for meat, milk, and eggs from livestock treated with antibiotics. Only 3.8 % of households indicted they always follow withdrawal period guidelines, either before selling or consuming animal products. This is consistent with studies in Ethiopia [47], Ghana [13], and Nigeria [30], where non-observance of withdrawal periods was linked to inadequate veterinary supervision, limited awareness, and informal drug distribution systems. In addition, studies in Ethiopia [28] and India revealed that farmers often prioritised immediate financial benefits over observing withdrawal periods and being concerned about residues of consumer risk. Moreover, self-treatment without appropriate veterinary guidance is common in many African settings, where veterinary services are scarce or unaffordable [2]. In this study, AMR knowledge was significantly associated with both improved access to veterinary services and higher satisfaction with veterinary care, reinforcing the role of extension and animal health systems in improving responsible antimicrobial use.

Education emerged as a strong and consistent predictor of knowledge on AMR. Respondents with primary, secondary, and post-secondary education were significantly more likely to report awareness of AMR. This reflects similar findings from Nigeria and India, where literacy and school attendance were linked to improved hygiene, food safety, and antimicrobial stewardship [16,32]. Higher asset ownership was also positively associated with knowledge and better practices, suggesting that wealthier households may have better access to services and information. Interestingly, religious affiliation influenced both knowledge and practice. Protestant and Pentecostal households were more likely to be aware of AMR and comply with withdrawal guidelines, while some faith groups, such as Apostolic sects, showed mixed effects. These findings are consistent with qualitative work in Southern Africa that shows religious beliefs influence health-seeking behaviour, especially in rural communities [18]. Geographic disparities were also notable as households in Matabeleland North consistently performed better in terms of both knowledge and compliance, while Mashonaland West and Matabeleland South performed poorly. This may reflect differential access to veterinary services, presence of NGOs, or historical investment in animal health systems [6].

It is important to view these findings in relation to the One Health perspective, which underscores the interconnectedness of human, animal and environmental health. Anti-microbial resistance is a clear example of a One Health issue because it affects people, animals, and the environment all at the same time [33]. The low level of awareness and practice of food safety observed in the present study suggests the need to integrate food safety with AMR awareness in training programs. Adopting the One Health framework in educating stakeholders about food safety if therefore important in Zimbabwe and beyond, as this can help strengthen measures to prevent the spread of AMR, particularly in informal food environments where regulations and oversight tend to be weak. This suggestion is consistent with calls from organizations such as the World Health Organization (WHO) and Africa Centres for Disease Control and Prevention (Africa CDC), which have emphasized the importance of multidiscipline collaboration as well as data and community awareness the improvements [40,43].

### 4.1 Implications for policy and practice

The strong link between education and AMR awareness suggests that targeted education campaigns can be impactful. Ministries of Health, Environment and Agriculture should mainstream AMR sensitisation and training into decentralised extension services, school curricula, and farmer field schools, prioritising districts with low uptake of awareness-raising. Simple, culturally adapted messages, which have been translated into the local languages, can be efficiently transmitted through community-based distribution systems such as Community Health Workers, agricultural extension officers, and para-veterinarians. These should also be based on the One Health approach, recognising the interface between human, animal, and environmental health, and allowing coordinated messaging on AMR, zoonoses, and food safety hazards.

In addition, policy tools are required to enforce drug withdrawal intervals to enhance food safety, with respect to the informal and smallholder livestock production contexts with less veterinary knowledge. Low-cost and quick testing kits of antibiotic residue. In addition, farmer cooperatives and producer groups can be induced to assume self-regulating roles through certification schemes, preferential market entry (premium market access), or subsidies for compliance. Finally, the informal food sector, which is the predominant route for the distribution of animal-sourced foods in much of rural and peri-urban settings, will need to be monitored and formalised. Not only would these efforts strengthen public health, but they would also increase consumer confidence, avoid food waste from rejected products, and support broader AMR containment and food system resilience objectives.

## 5 Conclusion

In conclusion, this study provides new evidence on the knowledge and practices surrounding AMR in livestock systems among rural households in Zimbabwe. The findings indicate widespread gaps in awareness and in adherence to recommended practices for antibiotic use and withdrawal periods. The study also identifies key demographic, socioeconomic, and geographic determinants that shape household-level behaviours, with significant implications for public health and food safety. This study provides nationally representative evidence on antimicrobial resistance (AMR) knowledge and antibiotic use practices among rural households in Zimbabwe. Results show that the level of awareness on AMR is alarmingly low and that a wide medium-low spread non-compliance to essential food safety behaviours is present, such observing antibiotic withdrawal periods in food animal production systems. Such practices have potentially serious public health implications, in countries where informal food systems are predominant. Level of education, socio-economic status, religion, disability status and geographic location were identified as key determinants of awareness and adherence, indicating the multilayered nature of behavioural change.

## CRediT authorship contribution statement

**George Kembo:** Writing – original draft, Methodology, Formal analysis, Conceptualization. **Lesley Macheka:** Writing – original draft, Methodology, Investigation, Formal analysis, Data curation, Conceptualization. **Mavis P. Dembedza:** Validation, Methodology, Formal analysis. **Desmond T. Mugadza:** Writing – review & editing, Validation, Formal analysis. **Faith A. Manditsera:** Writing – review & editing, Methodology, Investigation, Formal analysis.

## Funding

This research did not receive any specific grant from funding agencies in the public, commercial, or not-for-profit sectors.

## Declaration of competing interest

The authors declare no conflict of interest.

## Data Availability

Data will be made available on request.

## References

[1] Ahmed S.K., Hussein S., Qurbani K., Ibrahim R.H., Fareeq A., Mahmood K.A., Mohamed M.G. (2024). Antimicrobial resistance: impacts, challenges, and future prospects. J. Med. Surgery Public Health.

[2] Alhaji N.B., Isola T.O. (2018). Antimicrobial usage by pastoralists in food animals in north-Central Nigeria: the associated socio-cultural drivers for antimicrobials misuse and public health implications. One Health.

[3] Aslam B., Asghar R., Muzammil S., Shafique M., Siddique A.B., Khurshid M., Ijaz M., Rasool M.H., Chaudhry T.H., Aamir A., Baloch Z. (2024). AMR and sustainable development goals: at a crossroads. Glob. Health.

[4] Astivia O.L.O., Zumbo B.D. (2019). Heteroskedasticity in multiple regression analysis: what it is, how to detect it and how to solve it with applications in R and SPSS. Pract. Assess. Res. Eval..

[5] Bebell L.M., Muiru A.N. (2014).

[6] Catley A., Alders R.G., Wood J.L.N. (2012). Participatory epidemiology: approaches, methods, experiences. Vet. J..

[7] Caudell M.A., Quinlan M.B., Subbiah M., Call D.R., Roulette C.J., Roulette J.W., Roth A., Matthews L., Quinlan R.J. (2017). Antimicrobial use and veterinary care among Agro-pastoralists in northern Tanzania. PLoS One.

[8] Cochran W.G. (1942). Sampling theory when the sampling-units are of unequal sizes. J. Am. Stat. Assoc..

[9] D. Destoumieux-Garzón, P. Mavingui, G. Boetsch, J. Boissier, F. Darriet, P. Duboz, C. Fritsch, P. Giraudoux, F. Le Roux, S. Morand, C. Paillard, D. Pontier, C. Sueur and Y. Voituron, The one health concept: 10 years old and a long road ahead*.* Frontiers in veterinary Science, 2018. Volume 5–2018. 10.3389/fvets.2018.00014 PMC5816263 29484301

[10] Fuller W., Kapona O., Aboderin A.O., Adeyemo A.T., Olatunbosun O.I., Gahimbare L., Ahmed Y.A. (2023). Education and awareness on antimicrobial resistance in the WHO African region: a systematic review. Antibiotics.

[11] Grace D. (2015). Food safety in low and middle income countries. Int. J. Environ. Res. Public Health.

[12] Iskandar K., Molinier L., Hallit S., Sartelli M., Hardcastle T.C., Haque M., Lugova H., Dhingra S., Sharma P., Islam S., Mohammed I., Naina Mohamed I., Hanna P.A., Hajj S.E., Jamaluddin N.A.H., Salameh P., Roques C. (2021). Surveillance of antimicrobial resistance in low- and middle-income countries: a scattered picture*.* Antimicrob resist. Infect. Control..

[13] Johnson S., Bugyei K., Nortey P., Tasiame W. (2019). Antimicrobial drug usage and poultry production: case study in Ghana. Anim. Prod. Sci..

[14] Jorge P., Magalhães A.P., Grainha T., Alves D., Sousa A.M., Lopes S.P., Pereira M.O. (2019). Antimicrobial resistance three ways: healthcare crisis, major concepts and the relevance of biofilms. FEMS Microbiol. Ecol..

[15] Kallu S.A., Kebede N., Kassa T., Wubaye A.M., Kainga H., Mekonnen H., Simuunza M.C. (2024). Knowledge, attitudes, practices, and risk perception of antimicrobial use and antimicrobial resistance among dairy farm owners/workers in Addis Ababa, Ethiopia. Infect. Drug Resist..

[16] King S., Exley J., Taylor J., Kruithof K., Larkin J., Pardal M. (2016). Antimicrobial stewardship: the effectiveness of educational interventions to change risk-related behaviours in the general population: a systematic review. Rand Health Oper..

[17] Lemma M., Alemu B., Amenu K., Wieland B., Knight-Jones T. (2025). Enhancing community awareness of antimicrobial use and resistance through community conversations in rural Ethiopia. One Health Outlook.

[18] Lopes Ibanez-Gonzalez D., Tollman S.M. (2015). Clinics and churches: lifeworlds and health-seeking practices of older women with noncommunicable disease in rural South Africa. BMC Int. Health Hum. Rights.

[19] Luu Q.H., Nguyen T.L.A., Pham T.N., Vo N.G., Padungtod P. (2021). Antimicrobial use in household, semi-industrialized, and industrialized pig and poultry farms in Viet Nam. Prev. Vet. Med..

[20] Ma F., Xu S., Tang Z., Li Z., Zhang L. (2021). Use of antimicrobials in food animals and impact of transmission of antimicrobial resistance on humans. Biosaf. Health.

[21] Macheka L., Manditsera F.A., Ngadze R.T., Mubaiwa J., Nyanga L.K. (2013). Barriers, benefits and motivation factors for the implementation of food safety management system in the food sector in Harare Province, Zimbabwe. Food Control.

[22] Mugabe G.P., Kapungu F., Marimo S., Nys H., Knight-Jones T., Caron A., Richards S., Chirenda J. (2024). *O*ne health landscape in Zimbabwe: current status, challenges and opportunities for institutionalisation. One Health Cases.

[23] Mdegela R.H., Mwakapeje E.R., Rubegwa B., Gebeyehu D.T., Niyigena S., Msambichaka V., Nonga H.E., Antoine-Moussiaux N., Fasina F.O. (2021). Antimicrobial use, residues, resistance and governance in the food and agriculture sectors, Tanzania. Antibiotics.

[24] Melesse M.B., Tirra A.N., Homann-Kee Tui S., Van Rooyen A.F., Hauser M. (2023). Production decisions and food security outcomes of smallholder's livestock market participation: empirical evidence from Zimbabwe. Front. Sustainable Food Syst..

[25] Mshana S.E., Matee M., Rweyemamu M. (2013). Antimicrobial resistance in human and animal pathogens in Zambia, Democratic Republic of Congo, Mozambique and Tanzania: an urgent need of a sustainable surveillance system. Ann. Clin. Microbiol. Antimicrob..

[26] Mugadza D.T., Feresu K.W., Jombo T.Z., Mugombi J.W., Nyarugwe S.P., Chimuti S., Nyanhete V., Manditsera F.A., Macheka L. (2025). Food safety governance in Zimbabwe: challenges, regulatory gaps, and strategies for global compliance. Food Control.

[27] Murray C.J.L., Ikuta K.S., Sharara F., Swetschinski L., Aguilar G. Robles, Gray A., Han C., Bisignano C., Rao P., Wool E., Johnson S.C., Browne A.J., Chipeta M.G., Fell F., Hackett S., Haines-Woodhouse G., Hamadani B.H. Kashef, Kumaran E.A.P., McManigal B., Achalapong S., Agarwal R., Akech S., Albertson S., Amuasi J., Andrews J., Aravkin A., Ashley E., Babin F.-X., Bailey F., Baker S., Basnyat B., Bekker A., Bender R., Berkley J.A., Bethou A., Bielicki J., Boonkasidecha S., Bukosia J., Carvalheiro C., Castañeda-Orjuela C., Chansamouth V., Chaurasia S., Chiurchiù S., Chowdhury F., Donatien R. Clotaire, Cook A.J., Cooper B., Cressey T.R., Criollo-Mora E., Cunningham M., Darboe S., Day N.P.J., De Luca M., Dokova K., Dramowski A., Dunachie S.J., Bich T. Duong, Eckmanns T., Eibach D., Emami A., Feasey N., Fisher-Pearson N., Forrest K., Garcia C., Garrett D., Gastmeier P., Giref A.Z., Greer R.C., Gupta V., Haller S., Haselbeck A., Hay S.I., Holm M., Hopkins S., Hsia Y., Iregbu K.C., Jacobs J., Jarovsky D., Javanmardi F., Jenney A.W.J., Khorana M., Khusuwan S., Kissoon N., Kobeissi E., Kostyanev T., Krapp F., Krumkamp R., Kumar A., Kyu H.H., Lim C., Lim K., Limmathurotsakul D., Loftus M.J., Lunn M., Ma J., Manoharan A., Marks F., May J., Mayxay M., Mturi N., Munera-Huertas T., Musicha P., Musila L.A., Mussi-Pinhata M.M., Naidu R.N., Nakamura T., Nanavati R., Nangia S., Newton P., Ngoun C., Novotney A., Nwakanma D., Obiero C.W., Ochoa T.J., Olivas-Martinez A., Olliaro P., Ooko E., Ortiz-Brizuela E., Ounchanum P., Pak G.D., Paredes J.L., Peleg A.Y., Perrone C., Phe T., Phommasone K., Plakkal N., Ponce-de-Leon A., Raad M., Ramdin T., Rattanavong S., Riddell A., Roberts T., Robotham J.V., Roca A., Rosenthal V.D., Rudd K.E., Russell N., Sader H.S., Saengchan W., Schnall J., Scott J.A.G., Seekaew S., Sharland M., Shivamallappa M., Sifuentes-Osornio J., Simpson A.J., Steenkeste N., Stewardson A.J., Stoeva T., Tasak N., Thaiprakong A., Thwaites G., Tigoi C., Turner C., Turner P., van Doorn H.R., Velaphi S., Vongpradith A., Vongsouvath M., Vu H., Walsh T., Walson J.L., Waner S., Wangrangsimakul T., Wannapinij P., Wozniak T., Sharma T.E.M.W. Young, Yu K.C., Zheng P., Sartorius B., Lopez A.D., Stergachis A., Moore C., Dolecek C., Naghavi M. (2022). Global burden of bacterial antimicrobial resistance in 2019: a systematic analysis. Lancet.

[28] Mutua F., Kiarie G., Mbatha M., Onono J., Boqvist S., Kilonzi E., Mugisha L., Moodley A., Sternberg-Lewerin S. (2023). Antimicrobial use by peri-urban poultry smallholders of Kajiado and Machakos counties in Kenya. Antibiotics.

[29] Mutua F., Sharma G., Grace D., Bandyopadhyay S., Shome B., Lindahl J. (2020). *A* review of animal health and drug use practices in India, and their possible link to antimicrobial resistance. Antimicrob. Resist. Infect. Control.

[30] Njoga E.O., Ogugua A.J., Nwankwo I.O., Awoyomi O.J., Okoli C.E., Buba D.M., Oyeleye F.A., Ajibo F.E., Azor N., Ogunniran T.M. (2021). Antimicrobial drug usage pattern in poultry farms in Nigeria: implications for food safety, public health and poultry disease management. Vet. Ital..

[31] Peruzy M.F., Capuano F., Proroga Y.T.R., Cristiano D., Carullo M.R., Murru N. (2020). Antimicrobial susceptibility testing for Salmonella serovars isolated from food samples: five-year monitoring (2015–2019). Antibiotics.

[32] Popoola O.O., Adepitan D.S., Adeyemi A.S., Oladeru O.F., Yusuff S.I. (2024). A national survey of the antibiotic use, self-medication practices, and knowledge of antibiotic resistance among graduates of tertiary institutions in Nigeria. Sci. Afr..

[33] Robinson T.P., Bu D.P., Carrique-Mas J., Fèvre E.M., Gilbert M., Grace D., Hay S.I., Jiwakanon J., Kakkar M., Kariuki S., Laxminarayan R., Lubroth J., Magnusson U., Ngoc P. Thi, VanBoeckel T.P., Woolhouse M.E.J. (2016). Antibiotic resistance is the quintessential one health issue. Trans. R. Soc. Trop. Med. Hyg..

[34] Rodríguez-Varón A., Muñoz O.M., Pulido-Arenas J., Amado S.B., Tobón-Trujillo M. (2017). Antibiotic-associated diarrhea: clinical characteristics and the presence of Clostridium difficile. Revista de Gastroenterología de México (English Edition).

[35] Rware H., Monica K.K., Idah M., Fernadis M., Davis I., Buke W., Solveig D., Daniel K., Duncan C., Morten B., Keith H. (2024). Examining antibiotic use in Kenya: farmers' knowledge and practices in addressing antibiotic resistance. CABI Agric. Biosci..

[36] Sadiq M.B., Syed-Hussain S.S., Ramanoon S.Z., Saharee A., Ahmad N., Zin N.M., Khalid S., Naseeha D., Syahirah A., Mansor R. (2018). Knowledge, attitude and perception regarding antimicrobial resistance and usage among ruminant farmers in Selangor, Malaysia. Preventive Vet. Med..

[37] Salmon G., Teufel N., Baltenweck I., van Wijk M., Claessens L., Marshall K. (2018). Trade-offs in livestock development at farm level: different actors with different objectives. Glob. Food Sec..

[38] Sharma A., Singh A., Dar M.A., Kaur R.J., Charan J., Iskandar K., Haque M., Murti K., Ravichandiran V., Dhingra S. (2022). Menace of antimicrobial resistance in LMICs: current surveillance practices and control measures to tackle hostility. J. Infect. Public Health.

[39] Swiswa S., Pinto C.J., Chandler C.I. (2022).

[40] Varma J.K., Oppong-Otoo J., Ondoa P., Perovic O., Park B.J., Laxminarayan R., Peeling R.W., Schultsz C., Li H., Ihekweazu C. (2018). Africa centres for disease control and prevention's framework for antimicrobial resistance control in Africa. Afr. J. Lab. Med..

[41] WHO (2015).

[42] WHO (2022).

[43] WHO (2022).

[44] WHO (2022).

[45] Wooldridge J.M. (2016). Should instrumental variables be used as matching variables?. Res. Econ..

[46] World Medical Association (2013). World medical association declaration of Helsinki: ethical principles for medical research involving human subjects. JAMA.

[47] Woyessa M., Agga G.E., Gumi B., Ayana D., Mamo G. (2020). Antibiotic use in poultry production in selected districts of east Showa zone, Central Ethiopia: from antibiotic stewardship perspective. Am. Eurasian J. Sci. Res..

